# Anastomotic Leakage after Oesophagectomy: Upper Endoscopy or Computed Tomography First? Time Is of the Essence

**DOI:** 10.3390/diseases10040126

**Published:** 2022-12-14

**Authors:** Nader El-Sourani, Fadl Alfarawan, Sorin Miftode

**Affiliations:** University Hospital for General and Visceral Surgery, Klinikum Oldenburg AöR, Rahel-Strauss-Str. 10, 26133 Oldenburg, Germany

**Keywords:** oesophagectomy, anastomotic leakage, computed tomography, endoscopy

## Abstract

Introduction: Anastomotic leakage (AL) following oesophageal surgery is the most feared complication. Therefore, it is of utmost importance to diagnose it in a timely and safe manner. The diagnostic algorithm, however, differs across institutions world-wide, with no clear consensus or guidelines. The aim of this study was to analyse whether computed tomography (CT) or upper endoscopy (UE) should be performed first. Material and Methods: Records of 185 patients undergoing oesophageal surgery for underlying malignancy were analysed. All patients that developed an AL were further analysed. Results of CT and UE were compared to calculate sensitivity. Results: Overall, 33 out of 185 patients were diagnosed with an AL after oesophagectomy. All patients received a CT and a UE. The CT identified 23 out of 33 patients correctly. Sensitivity was 69.7% for CT, compared to 100% for UE. Conclusion: If patients are clinically suspicious regarding development of an AL after oesophagectomy, UE should be performed prior to CT as it has a sensitivity of 100%. In addition, treatment by means of endoluminal vacuum therapy (EVT) or self-expanding-metal stents (SEMS) can be initiated promptly.

## 1. Introduction

The incidence of oesophageal cancer in the European Union is around 43,700 cases per year and it is the 19th most common cancer in Europe [1]. Surgical resection after or without neoadjuvant therapy is indicated if a curative resection is possible. The most common procedure performed worldwide is the open or laparoscopic assisted abdominothoracic oesophageal resection with two-field lymph node dissection, and with an intrathoracic anastomosis based on Ivor-Lewis [2]. The stomach is the most commonly used substitute for reconstruction after oesophagectomy, resulting in an oesophagogastrostomy.

A postoperative AL is considered to be the most feared complication after surgery and is associated with an increased prolonged intensive care unit and hospital stay, increased hospital costs, decreased long-time survival and quality of life, and with a postoperative mortality rate ranging between 12–50% [3]. To combat this problem, many institutions perform a routine examination in the postoperative setting to rule out an AL. However, to this date, there are no international guidelines on whether a routine examination is useful and what imaging modality should be used. Hagens et al. questioned surgeons world-wide on their management of AL after oesophageal surgery in 2018 (13). In total, 62.8% responded that a routine examination was carried out, with a dynamic swallow study (DSS) being the most commonly performed (46.5%). Only 4.7% used computed tomography (CT) and 8.5% used upper endoscopy (UE).

However, available data on this subject over the last decade have shown that there is no clear benefit from a routine examination, with no impact on survival [4,5,6]. In addition, many institutions carried out a CT prior to a UE when an AL was suspected. Carrying out the wrong diagnostic modality can affect patient management and patient outcome. For instance, a non-viable conduit because of ischemia or necrosis can only be identified through UE. Therefore, the aim of our study was to evaluate which diagnostic modality should be the first choice when AL is suspected.

## 2. Material and Methods

### 2.1. Study Population

Between 2010 and 2022 all patients undergoing oesophageal surgery at our department were analysed. The data were extracted from a prospective data set and were retrospectively analysed. All patients that developed an AL after oesophagectomy were included in this study. All patients received a UE and CT. The following parameters were examined: sex, age, body mass index (BMI), American society of anaesthesiologist (ASA) classification, tumour location, tumour histology, R-Status, neoadjuvant therapy and surgical procedure. Approval by the Ethics Committee of the University of Oldenburg was obtained (2022-115-118).

### 2.2. Definition of an Anastomotic Leak

An AL is defined as a defect of the wall at the anastomotic site leading to communication between the intra- and extraluminal compartments [7]. In addition, severity of the leak should be graded I-III according to the ECCG classification. Grade I leaks require no intervention, grade II leaks require active intervention but no surgery and grade III leaks require surgical intervention [8].

### 2.3. Computed Tomography

**Figure 1:**
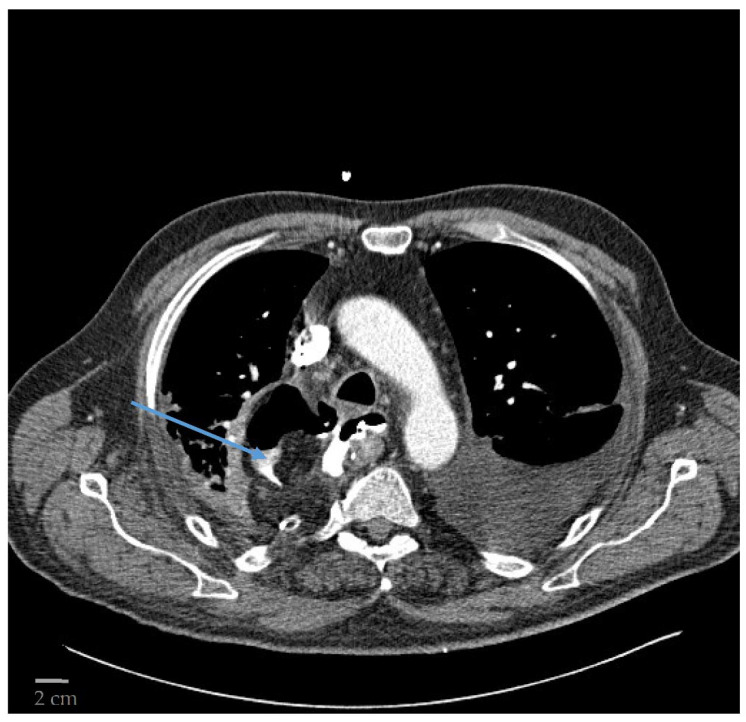
CT displaying an AL with extravasation of contrast agent and free air in the mediastinum indicated by the blue arrow.

The Canon Aquilion One ^®^ was used in all patients. It has 100 kW of power with a gantry bore of 78 cm and a scan range of 150/200 cm. It rotates at 0.350 s per rotation with a maximum rate of 50 images per second delivered. CT was performed with intravenous and oral contrast agents. Patients were in a supine position and images were acquired while in full inspiration. A 1.5 mL/kg contrast agent was infused intravenously. In addition, 15 mL of contrast agent mixed with 200 mL of water were given orally. Axial images were obtained in slice thicknesses of 3 mm. Extravasation of the contrast agent was defined as an AL. In addition, mediastinal fluid collection and mediastinal air were associated with the presence of an AL (Figure 1). All CT images were reviewed by an experienced radiologist (specialist/consultant).

### 2.4. Upper Endoscopy

**Figure 2:**
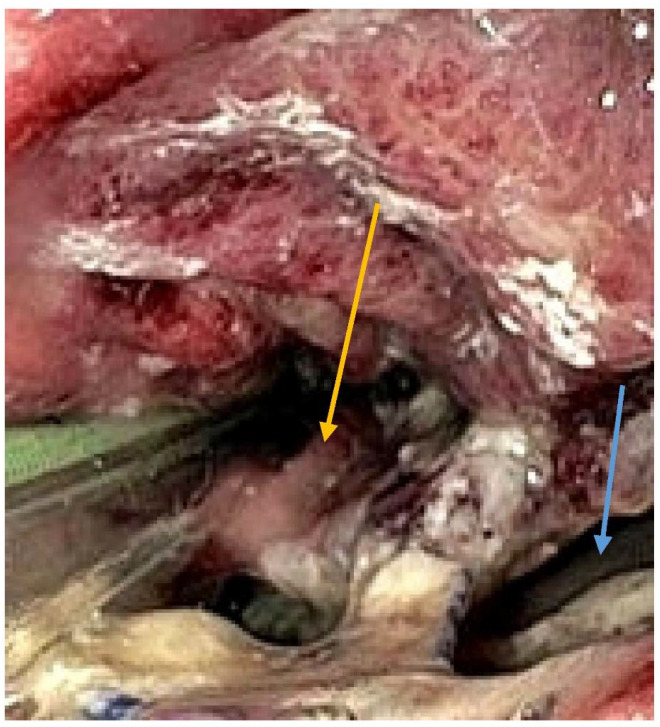
Endoscopy showing a circumferential anastomotic leakage after oesophagectomy indicated by the blue arrow. Yellow arrow is pointing to the true lumen. The radiologist and the endoscopist were blinded to their respective findings.

UE was performed to evaluate the conduit and the anastomosis after oesophageal surgery. A small-calibre nasal endoscope (GIF-N180) was used. It provides excellent visibility and can detect a malperfused or necrotic conduit. In addition, endoscopy can be safely used in ventilated and neurologically deficient patients. Apart from the integrity of the anastomosis, abnormalities of the gastric conduit such as necrosis and ischemia were also documented. AL was defined as endoscopic visualisation of a breakdown of the anastomosis (Figure 2). Also, treatment such as a self-expanding metal stent (SEMS) or endoluminal vacuum therapy (EVT) can be initiated. All UE were performed by an experienced gastroenterologist (specialist/consultant).

### 2.5. Statistical Analysis

The statistical analysis was performed with IBM SPSS Statistics Version 64-Bit Version for Mac OS. Continuous variables were expressed as medians.

## 3. Results

### 3.1. Patient Demographics

Out of 185 patients a total of 33 developed an AL after oesophagectomy, yielding an insufficiency rate of 17.8%. All patients received a combination of a CT with oral and intravenous contrast and a UE when a leak was clinically suspected. Patient demographics, tumour characteristics and surgical procedure are shown in Table 1. The median age was 58.5 years (range: 32–83). Males were more likely affected than females with a median BMI of 25.5 (range: 16–47).

### 3.2. Computed Tomography

CT was performed in all 33 patients who had clinical signs suspicious of an AL. An AL was correctly diagnosed in 23 patients. In 10 patients no signs of an AL were described, yielding a sensitivity of 69.7% (Table 2).

### 3.3. Upper Endoscopy

UE was performed in all 33 patients who had clinical signs suspicious of an AL. UE confirmed anastomotic leakages in all patients who were clinically suspicious. In addition, it diagnosed ten leaks that were missed by CT, yielding a sensitivity of 100% (Table 2).

### 3.4. Anastomotic Leakage

AL was diagnosed in a total of 33 patients. According to the ECCG classification there were three grade I leaks, twenty-six grade II leaks and four grade III leaks. The majority of the patients were treated by EVT (n = 17), followed by SEMS (n = 8) and Clipping (n = 1). Three patients were treated conservatively by nil per mouth, antibiotics and an enteral feeding tube, while four patients required redo-surgery. The overall mortality rate in patients with AL was 30.3%.

## 4. Discussion

Following oesophageal surgery, the status of the intrathoracic anastomosis is of utmost importance, as an insufficiency dramatically increases morbidity and mortality and, therefore, leads to an increased intensive care unit stay, increases hospital stay and has a major impact on the long-time survival of the patient [9,10,11]. A gold standard for the diagnosis of an AL does not exist. To this day, many institutions still discuss the value of dynamic swallow studies in detecting an AL, although experiences and our own data show that the image modality has a very low sensitivity and a high false negative ratio [3].

Hagens et al. carried out an international survey on the management of AL after oesophagectomy in 2018 [12]. Of all 129 responders, 62.8% still use imaging techniques postoperatively on a routine basis, with a dynamic swallow study (46.5%) being the most used modality, followed by a chest x-ray (42.6%), UE (8.7%) and CT (4.7%). In addition, the surgeons were questioned on their first and second choices of diagnostic modalities in case of suspected AL. Over 40% used CT as their primary diagnostic tool, compared to 22% who used UE. It is clear that the optimal diagnostic modality for an AL after oesophagectomy remains controversial. However, our results show that UE correctly identified all AL when AL was suspected. In contrast, CT misdiagnosed 10 patients as having no AL, thus delaying correct patient management with possible serious consequences. The sensitivity to detect AL correctly was 69.7% in our patient collective as shown in Table 2. Similar results have been described by Song et al. They performed CT and UE as routine tools in all of their 71 patients and described a sensitivity of 71.4% with CT, compared to 100% with UE [13]. UE should be preferred over CT as the first choice of diagnostic modalities in case of suspected AL. In addition to its superior sensitivity, UE is the only diagnostic modality that has the ability to determine the viability of the gastric conduit. In cases of severe ischemia and/or necrosis, redo-surgery becomes necessary.

The limitations of our study are that it is a retrospective analysis, and it is from a single institution. Patient selection bias might be inevitable because only patients that had a proven AL were enrolled. However, the main question of the study was whether UE or CT should be the first choice when AL is suspected.

In conclusion, detecting an AL is of utmost importance as it severely impacts the postoperative outcome of the patient. Therefore, we can recommend performing a UE prior to a CT. In addition, UE has the ability to accurately determine the viability of the gastric conduit and to directly treat the AL by means of EVT or SEMS. A routine examination after surgery is not recommended.

## Figures and Tables

**Table 1:** Patient and tumour characteristics.

Variables	
Male	28 (84.8%)
Female	5 (15.2%)
Age	58.5 (32–83)
BMI ^a^	25.5 (16–47)
ASA ^b^ I	1 (3%)
ASA II	18 (54.5%)
ASA III	14 (42.5%)
Tumour location	
Middle third	1 (3%)
Distal third	32 (97%)
Tumour histology	
Adenocarcinoma	30 (91%)
Squamous cell carcinoma	3 (9%)
Open procedure	28 (84.8%)
Hybrid procedure	5 (15.2%)
R0-Resection	33 (100%)
Chemotherapy	16 (48.5%)
Radiochemotherapy	5 (15.2%)
No neoadjuvant therapy	12(36.3%)

^a^ Body Mass Index, ^b^ American Society of Anesthesiologists.

**Table 2:** Efficiency and Feasibility of UE and CT.

	No	UE	CT
Leaks	33	33	23
		0	10
Sensitivity		100 %	69.7%
Technical feasibility	33	33	33

## Data Availability

The data presented in this study are available on request from the corresponding author. The data are not publicly available due to data management of the hospital.

## References

[1] Stahl M., Budach W., Meyer H.J., Cervantes A., ESMO Guidelines Working Group (2010). Esophageal cancer: Clinical Practice Guidelines for diagnosis, treatment and follow-up. Ann. Oncol..

[2] Siewert J.R. (2007). Osophaguskarzinom [Esophageal Carcinoma]. Der Chirurg..

[3] El-Sourani N., Bruns H., Troja A., Raab H.R., Antolovic D. (2017). Routine Use of Contrast Swallow after Total Gastrectomy and Esophagectomy: Is It Justified?. Pol. J. Radiol..

[4] Fan S.T., Lau W.Y., Yip W.C., Poon G.P., Yeung C., Wong K.K. (1988). Limitations and dangers of gastrografin swallow after esophageal and upper gastric operations. Am. J. Surg..

[5] Griffin S.M., Lamb P.J., Dresner S.M., Richardson D.L., Hayes N. (2001). Diagnosis and management of a mediastinal leak following radical oesophagectomy. Br. J. Surg..

[6] Hölscher A.H., Vallböhmer D., Brabender J. (2006). The prevention and management of perioperative complications. Best Pract. Res. Clin. Gastroenterol..

[7] Rahbari N.N., Weitz J., Hohenberger W., Heald R.J., Moran B., Ulrich A., Holm T., Wong W.D., Tiret E., Moriya Y. (2010). Definition and grading of anastomotic leakage following anterior resection of the rectum: A proposal by the International Study Group of Rectal Cancer. Surgery.

[8] Koch M., Garden O.J., Padbury R., Rahbari N.N., Adam R., Capussotti L., Fan S.T., Yokoyama Y., Crawford M., Makuuchi M. (2011). Bile leakage after hepatobiliary and pancreatic surgery: A definition and grading of severity by the International Study Group of Liver Surgery. Surgery.

[9] Low D.E., Kuppusamy M.K., Alderson D., Cecconello I., Chang A.C., Darling G., Davies A., D’Journo X.B., Gisbertz S.S., Griffin S.M. (2019). Benchmarking Complications Associated with Esophagectomy. Ann. Surg..

[10] Lagarde S.M., de Boer J.D., ten Kate F.J., Busch O.R., Obertop H., van Lanschot J.J. (2008). Postoperative complications after esophagectomy for adenocarcinoma of the esophagus are related to timing of death due to recurrence. Ann. Surg..

[11] Rizk N.P., Bach P.B., Schrag D., Bains M.S., Turnbull A.D., Karpeh M., Brennan M.F., Rusch V.W. (2004). The impact of complications on outcomes after resection for esophageal and gastroesophageal junction carcinoma. J. Am. Coll. Surg..

[12] Palmes D., Brüwer M., Bader F.G., Betzler M., Becker H., Bruch H.P., Büchler M., Buhr H., Ghadimi B.M., Hopt U.T. (2011). Diagnostic evaluation, surgical technique, and perioperative management after esophagectomy: Consensus statement of the German Advanced Surgical Treatment Study Group. Langenbecks Arch. Surg..

[13] Hagens E.R.C., Anderegg M.C.J., van Berge Henegouwen M.I., Gisbertz S.S. (2018). International Survey on the Management of Anastomotic Leakage after Esophageal Resection. Ann. Thorac. Surg..

